# Forming heterojunction: an effective strategy to enhance the photocatalytic efficiency of a new metal-free organic photocatalyst for water splitting

**DOI:** 10.1038/srep29327

**Published:** 2016-07-29

**Authors:** Hengshuai Li, Haiquan Hu, Chunjiang Bao, Feng Guo, Xiaoming Zhang, Xiaobiao Liu, Juan Hua, Jie Tan, Aizhu Wang, Hongcai Zhou, Bo Yang, Yuanyuan Qu, Xiangdong Liu

**Affiliations:** 1 School of Mechanical & Automotive Engineering, Liaocheng University, Liaocheng, 252059, China; 2 School of Physics and State Key Laboratory of Crystal Materials, Shandong University, Jinan 250100, China; 3 School of Physics Science and Information Technology, Liaocheng University, Liaocheng, 252059, China

## Abstract

Photocatalytic water splitting is a new technology for the conversion and utilization of solar energy and has a potential prospect. One important aspect of enhancing the photocatalytic efficiency is how to improve the electron-hole separation. Up to now, there is still no ideal strategy to improve the electron-hole separation. In this article, for metal-free organic photocatalysts, we propose a good strategy- forming heterojunction, which can effectively improve the electron-hole separation. We provide a metal-free organic photocatalyst g-C_12_N_7_H_3_ for water splitting. The stability of g-C_12_N_7_H_3_ has been investigated, the X-ray diffraction spectra has been simulated. Using first-principles calculations, we have systematically studied the electronic structure, band edge alignment, and optical properties for the g-C_12_N_7_H_3_. The results demonstrated that g-C_12_N_7_H_3_ is a new organocatalyst material for water splitting. In order to enhance the photocatalytic efficiency, we provided four strategies, i.e., multilayer stacking, raising N atoms, forming g-C_9_N_10_/g-C_12_N_7_H_3_ heterojunction, and forming graphene/g-C_12_N_7_H_3_ heterojunction. Our research is expected to stimulate experimentalists to further study novel 2D metal-free organic materials as visible light photocatalysts. Our strategies, especially forming heterojunction, will substantially help to enhance the photocatalytic efficiency of metal-free organic photocatalyst.

In the modern world, the excessive utilization of fossil fuels has brought two major issues: energy crisis and environmental pollution. Hence looking for a clean and renewable energy source is an effective method to solve the two issues. Hydrogen is considered to be the very promising energy. Hydrogen as the carrier of energy can avoid the environmental problem brought by utilization of traditional fossil fuels, because the production is water after hydrogen releasing the chemical energy. Photocatalysis technique is to use the photogenerated electrons and holes after absorbing the sunlight of the semiconductor-based photocatalyst to split water into H_2_ and O_2_^1^. Thus photocatalytic water splitting is a good method for the conversion and utilization of solar energy and has a potential prospect^2^.

Honda and Fujishima Obtained the Hydrogen through TiO_2_ for the first time^3^. To date, various oxide, sulfide, and oxynitride semiconductor photocatalysts have been developed for the aforementioned photocatalytic reaction^4^,^5^,^6^,^7^,^8^,^9^,^10^,^11^,^12^. These materials are basically inorganic. Inorganic photocatalyst has some disadvantages, such as limited concentration of active sites, toxicity of heavy metals^13^,^14^. In sharp contrast, organic photocatalyst has many advantages, such as low cost, easy fabrication, and mechanical flexibility^15^,^16^. So the development of metal-free organic efficient photocatalytic materials is a significant scientific research task.

Recently, Wang *et al*. reported that a metal-free organic polymeric photocatalyst, graphitic carbon nitride (g-C_3_N_4_), showed a good photocatalytic performance for hydrogen or oxygen production via water splitting^17^. The metal-free g-C_3_N_4_ photocatalysts possess very high thermal and chemical stability as well as interesting electronic properties, which make them valuable materials for photocatalysis-driven applications. Recently, many strategies, such as heteroatom doping^18^,^19^, multilayer stacking^20^, metal co-catalysts^21^,^22^, the hybrid complex^23^, and adsorbed dyes^24^ have been proposed to enhance its photocatalytic properties.

Because of the structure and properties similar to g-C_3_N_4_, the two-dimensional covalent triazine frameworks (2D-CTFs) have begun to enter the field of scientists. The 2D-CTFs can be synthesized by trimerization reaction of carbonitriles and adopted triazine ring (C_3_N_3_H_3_) as the building units^25^,^26^,^27^. Zhao *et al*. analyzed the electronic structure and optical absorption properties of CTF-0, CTF-1, and CTF-2, and gave three strategies to reduce the band gap^28^. There are still many structures of the 2D-CTFs that need to be studied, and many photocatalytic mechanisms that need to be explored.

As we all know, the progress of the photocatalytic water splitting contains three crucial steps: solar light harvesting, electron-hole separation and transportation, and the catalytic H_2_ and O_2_ evolution reactions. In the second step of electron-hole separation and transportation, one important aspect of enhancing the photocatalytic efficiency is how to reduce the electron-hole recombination, in other words, that is how to improve the electron-hole separation. Up to now, there is no method that can completely prohibit the electron-hole recombination, and there is still no ideal strategy to improve the electron-hole separation. In this article, for metal-free organic photocatalysts, we propose a very good strategy-forming heterojunction, which can effectively improve the electron-hole separation.

Here, we predict another 2D-CTF organic photocatalytic material consisted of benzene rings and triazines. These units are connected by C-C bonds resulting in a graphene-like carbon nitride with a chemical composition of C_12_N_7_H_3_ (referred to as g-C_12_N_7_H_3_). From first-principles, the calculations using the HSE06 function^29^ have been carried out to accurately predict the structural, energy band, redox potentials and the imaginary part of the dielectric function. In order to enhance the efficiency of the photolysis, we proposed four strategies including multilayer stacking, raising nitrogen, forming g-C_9_N_10_/g-C_12_N_7_H_3_ heterojunction, and forming graphene/g-C_12_N_7_H_3_ heterojunction. Need to point out that, forming heterojunction is an effective strategy, which can improve the electron-hole separation, so as to enhance the photocatalytic efficiency.

## Results and Discussion

**Figure 1:**
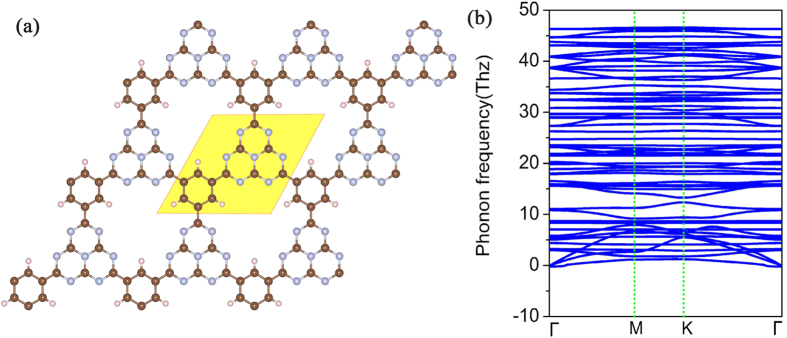
Schematic representation of g-C_12_N_7_H_3_ with the unit cells indicated by the light yellow region (**a**); the corresponding phonon spectrums along highly symmetric points in BZ are plotted in (**b**).

Figure 1(a) gives the optimized configuration of g-C_12_N_7_H_3_ lattice. Different from g-C_3_N_4_, the triazines and benzene rings in g-C_12_N_7_H_3_ are joined together via C-C bonds without the additional N atoms, resulting in a honeycomb lattice with a chemical formula of C_12_N_7_H_3_ per primitive cell. The six-fold symmetry and planar configuration are well kept in g-C_12_N_7_H_3_ structure. The C-N bond length in the triazine rings is 1.41 Å, a little longer than that in g-C_3_N_4_ (1.327 Å). The C-C bond length between the triazines and benzene rings is 1.48 Å, which is slightly longer than that in graphene (1.42 Å). This may be related to the polarization of the dangling bond electrons by the local strain and quantum entrapment of the core and bonding electrons at the edges^30^.

We tested the stability of the g-C_12_N_7_H_3_ framework using two methods. Firstly, we calculated the phonon dispersion curves of the g-C_12_N_7_H_3_ structure to test its kinetic stability. The phonon dispersion curves were calculated by the force-constant theory combined with the VASP code. The phonon spectrum along high-symmetric directions in BZ was shown in Fig. 1(b). Obviously, we did not find the modes with imaginary frequencies in the phonon spectrum along the highly symmetric points in BZ, and thus conclude they are dynamically stable. Secondly, we performed molecular dynamics (MD) simulations to test the dynamic stability of g-C_12_N_7_H_3_. A large (3 × 3) supercell containing 198 atoms is simulated with a Nose–Hoover thermostat at 300 K. After 30 ps, we found that the structure remains unchanged, which indicates the stability of g-C_12_N_7_H_3_ at room temperature. This can be understood by the fact that the binding energies of the C-N, C-C and C-H bonds are much larger than the thermal energy corresponding to room temperature^31^.

**Figure 2:**
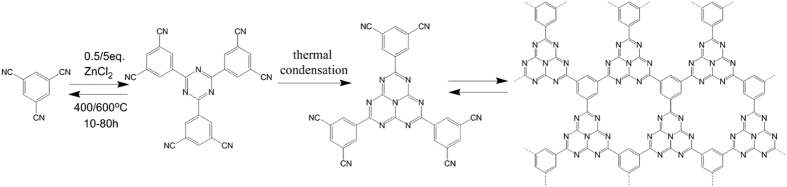
Schematic illustration of the trimerization of 1,3,5-tricyanobenzene in molten ZnCl_2_.

In the recent experiments, some similar structures have been synthesized^25^,^26^,^27^, which prove that the synthesis of g-C_12_N_7_H_3_ is convincing. After discussions and analyses with a number of experimental scientists, we gave the synthesis process and the synthesis method, which is shown in Fig. 2. In the first step, CTF-0 was synthesized under ionothermal conditions in sealed quartz ampules by heating 1,3,5-tricyanobenzene (TCB) in the presence of ZnCl_2_^25^. In the second step, we can produce g-C_12_N_7_H_3_ by the thermal condensation of the CTF-0. The recent works have shown that indeed the thermal condensation of cyanamide, dicyandiamide, or melamine yields a melon polymer built up from melem units^32^,^33^,^34^, confirming that the tri-s-triazine rings can be obtained by the thermal condensation of s-Triazine structure. Up to now, the thermal condensation pathways of cyanamide to dicyandiamide and later to melamine and all of the other C/N materials were seen as good synthetic strategies to generate slightly defect, polymeric species^35^,^36^. So the experiment process is reasonable, and it can be realized completely.

**Figure 3:**
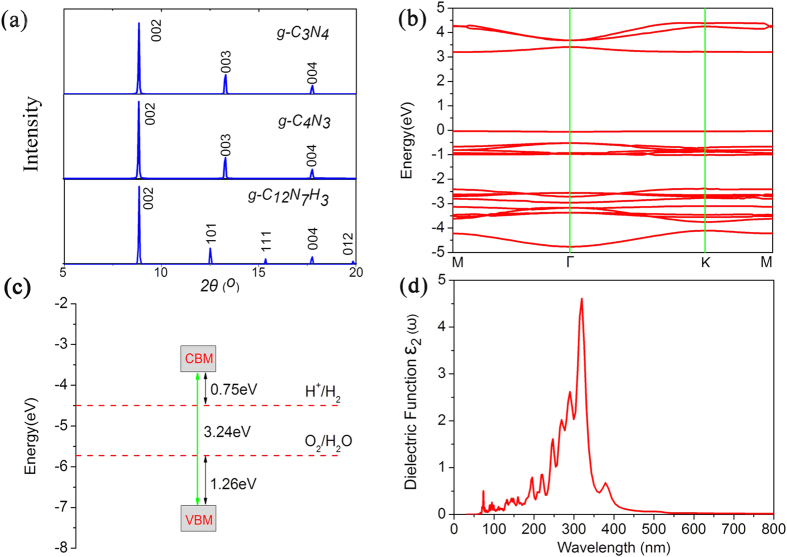
(**a**) Simulated XRD patterns of the g-C_12_N_7_H_3_. (**b**) Calculated band dispersion of the g-C_12_N_7_H_3_ with HSE06 methods; the valence band maximum is set to zero. (**c**) Band alignments of the g-C_12_N_7_H_3_ with respect to the standard water redox potentials. (**d**) Calculated imaginary part of the dielectric function (in-plane polarization) for the g-C_12_N_7_H_3_ with HSE06 methods.

The X-ray diffraction (XRD) spectra are quite useful for the experimental characterization of novel materials. We therefore simulated the XRD spectrum of g-C_12_N_7_H_3_ with a wavelength of 1.540562 Å [Fig. 3(a)]. For comparison, the XRD spectra of the already-synthesized g-C_3_N_4_^37^ and g-C_4_N_3_ lattices were also presented. It is clear that the g-C_12_N_7_H_3_ have a sharp peak near 2***θ*** = 8.85°and 17.72°, agreeing with the g-C_3_N_4_, g-C_4_N_3_ and g-C_9_N_7_^38^,^39^. However, g-C_12_N_7_H_3_ has additional peaks at 2***θ*** = 12.5°, 15.33°, and 19.83°, which can be regarded as fingerprints to identify the g-C_12_N_7_H_3_ from other graphitic carbon nitride materials.

We discussed whether the g-C_12_N_7_H_3_ is suitable as photocatalyst for water splitting according to the electronic structures. The calculated energy band gap is 2.3 eV at PBE level. As we all known, the general gradient approximation PBE will significantly underestimate the size of the band gap. A hybrid functional such as HSE06^29^ is expected to perform very well in predicting the accurate gap. So we calculated the energy band gap using HSE06 hybrid function. The more accurate gap is 3.24 eV. As shown in Fig. 3(b), because both the conduction band minimum (CBM) and valence band maximum (VBM) are situated on M point, so the g-C_12_N_7_H_3_ is a direct band gap semiconductor. The band gap of HSE06 is 0.94 eV larger than the PBE level, it still located at the marginal visible light region.

Another important aspect that decides their performance in photocatalytic water splitting is the band edge potential of photocatalysts. An ideal photocatalyst for water splitting would have certain important characteristics in its electronic structure. The positions of the conduction and valence band edges should straddle the redox potentials for water photolysis^40^,^41^,^42^. This means that the conduction band edge should be above the energy corresponding to the water reduction potential [*φ*(H^+^/H_2_)], and the valence band edge should be below the energy of the water oxidation potential [*φ*(O_2_/H_2_O)], in other words, the band gap must be wider than 1.23 eV, which is the difference between the water reduction potential and water oxidation potential. From Fig. 3(c), the band gap of the g-C_12_N_7_H_3_ is 3.24 eV, with suitable positions of the CBM and the VBM (E_CBM_ = 0.75 eV and E_VBM_ = 1.26 eV vs. NHE, pH = 0). Therefore, the electron at the lowest unoccupied molecular orbital (LUMO) is sufficiently reactive to reduce water to hydrogen, meanwhile the hole at the highest occupied molecular orbital (HOMO) has enough reduction potential to oxidize water to oxygen.

In order to study the optical absorption properties of g-C_12_N_7_H_3_, we calculated the frequency-dependent dielectric matrix with the hybrid HSE06 function. The complex dielectric constants at a given frequency can be defined as


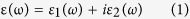


The expression for the absorption coefficient I(ω) was given as^43^:


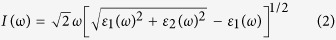


on the basis of the equation, the light absorbing information can be gained from the value of the imaginary part. The absorption coefficient was above zero, when only if the imaginary part


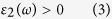


The imaginary part is determined by a summation over empty states using the equation


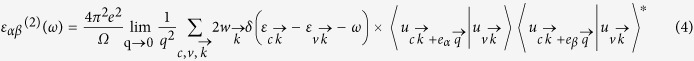


where the indices c and v represent conduction and valence band states, and 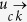 is the cell periodic part of the orbitals at the k point. A large number of empty conduction band states, which is almost twice more than the number of valence band, are included for the summation of equation (4).

The imaginary part of the dielectric function for the g-C_12_N_7_H_3_ is calculated using HSE06 hybrid function and shown in Fig. 3(d). We can test whether the structure can have efficient visible-light absorption. Obviously, the g-C_12_N_7_H_3_ is capable of harvesting visible light from Fig. 3(d). To sum up the characteristics of g-C_12_N_7_H_3_, it is a semiconductor, possesses suitable positions of the CBM and the VBM, and has the ability to absorb visible light, so it can be used as a photocatalyst for water splitting. However, it can only absorb a small portion of the visible light, resulting to low photoconversion efficiency. Therefore, the remaining question is how to enhance the photocatalytic efficiency.

The first strategy is multilayer stacking. The work functions and the energy band gap of two-, three-, four-, five-, and six-layer g-C_12_N_7_H_3_ computed with HSE06 methods are listed in Table 1. As a comparison, the results calculated by the PBE methods are shown in the Supplementary Information. When the number of layers increases, the work function is basically stable. However, the band gap decreases gradually with the increase of the number of layers. Analogous to the monolayer, all multilayer g-C_12_N_7_H_3_ are still direct gap semiconductor. When the numbers of layers are 2–6, the band gaps are 2.91–2.61 eV, respectively. With the increase of the number of layers, the material is close to a bulk material, which band gap is also 2.61 eV. Compared with monolayer, the band gap decreases 0.63 eV by multilayer stacking.

**Figure 4:**
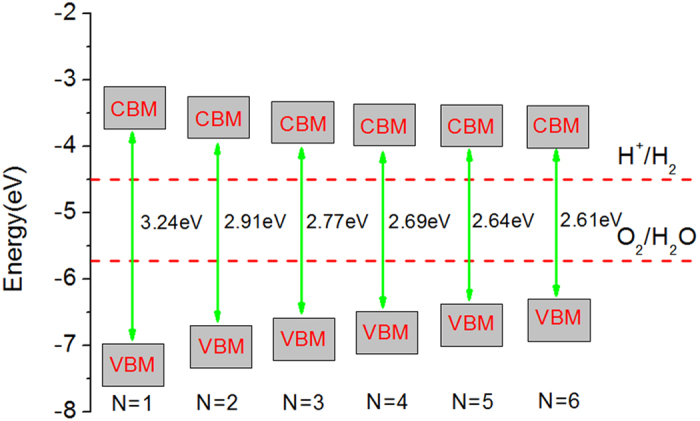
Band alignments of n-layers g-C_12_N_7_H_3_ relative to the standard water redox potentials. The reference potential is the vacuum level.

In order to better analyze the photocatalytic ability of multilayer g-C_12_N_7_H_3_, the band edge potential of multilayer g-C_12_N_7_H_3_ are shown in Fig. 4. Relative to the monolayer, the VBM of the bilayer system rose, meanwhile the CBM of the bilayer system declined. The other multilayer g-C_12_N_7_H_3_ systems are similar to the bilayer system. The multilayer g-C_12_N_7_H_3_ systems, possessing suitable band edges, are able to split water with simultaneous evolution of hydrogen and oxygen.

**Figure 5:**
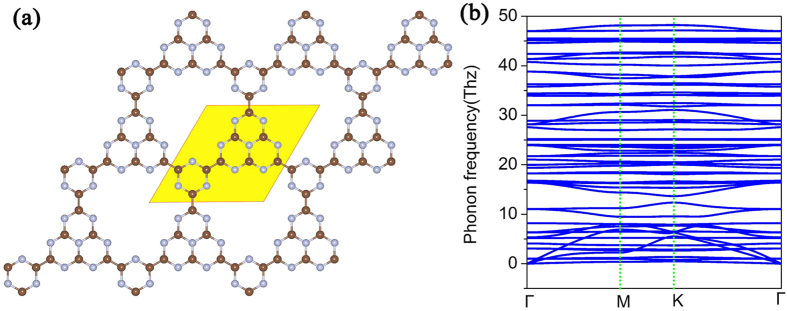
Schematic representation of g-C_9_N_10_ with the unit cells indicated by the light yellow region (**a**); the corresponding phonon spectrums along highly symmetric points in BZ are plotted in (**b**).

The second strategy is raising N atoms. In recent years, many experiments have already proved that the photocatalytic efficiency is obviously enhanced by N doping in carbon-based photocatalysts^44^,^45^,^46^,^47^,^48^,^49^. Actually, some N-doped carbon nanomaterials were found to be excellent metal-free catalysts for water splitting^44^,^45^. We propose another 2D-CTF material of carbon nitride using C_9_N_10_ units as building blocks. These units are connected via C-C bonds resulting in a graphene-like carbon nitride with a stoichiometry of C_9_N_10_ (referred to as g-C_9_N_10_). The structure of g-C_9_N_10_ was shown in Fig. 5(a). Compare with Figs 1(a) and 5(a), the two structures are similar, but the content of N atoms is increasing in g-C_9_N_10_. In order to verify the stability of the structure, the phonon spectrum of the g-C_9_N_10_ has been calculated, as shown in Fig. 5(b). There is no imaginary frequencies in the phonon spectrum along the highly symmetric points in BZ, and thus conclude they are dynamically stable. Meanwhile, we performed molecular dynamics (MD) simulations to test the stability of the structure at room temperature. The synthesis of the g-C_9_N_10_ can refer to the synthesis process of the g-C_12_N_7_H_3._

**Figure 6:**
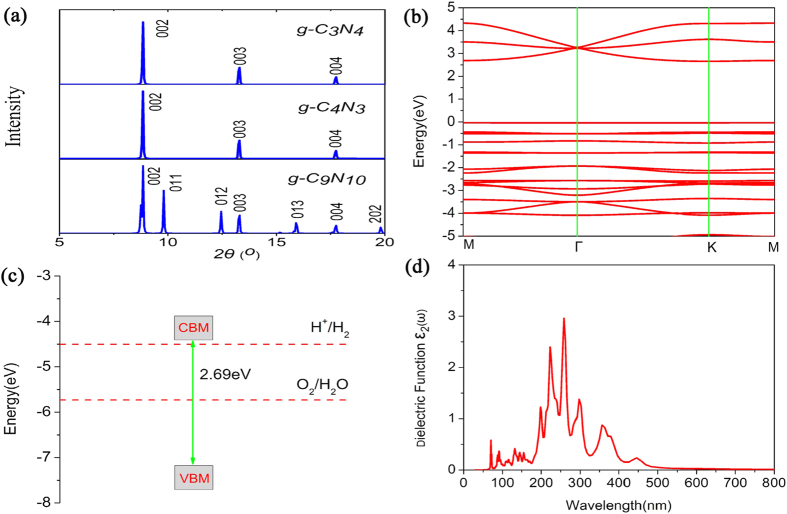
(**a**) Simulated XRD patterns of the g-C_9_N_10_. (**b**) Calculated band dispersion of the g-C_9_N_10_ with HSE06 methods; the valence band maximum is set to zero. (**c**) Band alignments of the g-C_9_N_10_ with respect to the standard water redox potentials. (**d**) Calculated imaginary part of the dielectric function (in-plane polarization) for the g-C_9_N_10_ with HSE06 methods.

The simulated XRD patterns of the g-C_9_N_10_ have been plotted in Fig. 6(a). It is clear that the g-C_9_N_10_ have a sharp peak near 2***θ*** = 8.85 °, 13.27° and 17.72°, agreeing with the g-C_3_N_4_, g-C_4_N_3_. Moreover, g-C_9_N_10_ has additional peaks at 2***θ*** = 9.79°, 12.5°, 15.91° and 19.83°. Its own special peak helps to distinguish it from a lot of similar structures.

The band structure of the g-C_9_N_10_ was calculated using the HSE06 function, is shown in Fig. 6(b). The energy band gap is 2.69eV. The decrease of band gap is distinct with respect to the g-C_12_N_7_H_3._ From Fig. 6(c), we can know that the g-C_9_N_10_ was able to split water with simultaneous evolution of hydrogen and oxygen in the presence of electron donors and acceptors. Figure 6(d) is calculated imaginary part of the dielectric function (in-plane polarization) for the g-C_9_N_10_ with the HSE06 function. The absorbing range is enlarged, including more visible light. Thus it can be seen that raising N atoms will enlarge the absorbing range and enhance the photocatalytic efficiency.

**Figure 7:**
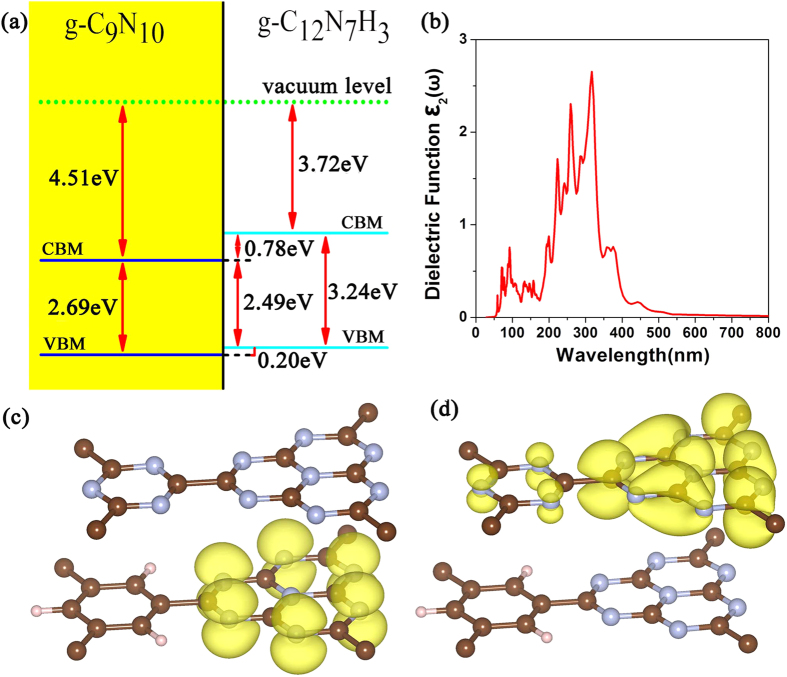
(**a**) The level of the valence band and conduction band of the g-C_9_N_10_/g-C_12_N_7_H_3_ heterojunction. (**b**) Calculated imaginary part of the dielectric function (in-plane polarization) for the g-C_9_N_10_/g-C_12_N_7_H_3_ heterojunction with HSE06 methods. The charge distribution of the valence band maximum (VBM) (**c**) and conduction band minimum (CBM) (**d**) with an isovalue of 0.002 e Å^−3^.

The third strategy is forming g-C_9_N_10_/g-C_12_N_7_H_3_ heterojunction. In this case, two semiconductors formed heterojunction, its energy level of the valence band and conduction band was shown in Fig. 7(a). Because of the selection rules of electronic transition, the change of optical absorption spectrum is limited. We can also get the results from comparison between Figs 6(d) and 7(b). However, the significant value of forming the heterojunction is that the electron-hole separation has been greatly improved. The charge distribution of the valence band maximum (VBM) and conduction band minimum (CBM) are presented in Fig. 7(c,d). The VBM is mainly composed of the p_z_ orbitals of nitrogen atoms in g-C_12_N_7_H_3_, however the CBM is composed of the the p_z_ orbitals of nitrogen and carbon atoms in g-C_9_N_10_. The charge density distributions of VBM and CBM have no overlap, which indicated that the g-C_9_N_10_/g-C_12_N_7_H_3_ heterojunction can efficiently separate the electrons and holes. The efficient electron-hole separation is conducive to enhance the photocatalytic efficiency.

**Figure 8:**
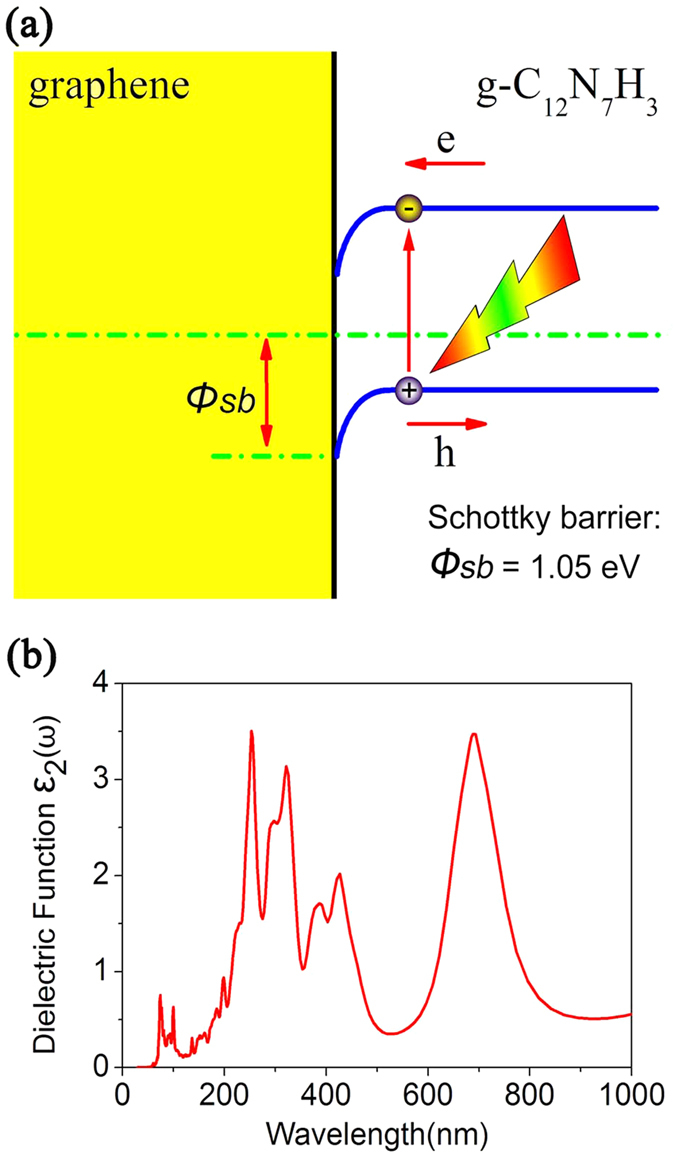
(**a**) Band alignment at graphene/g-C_12_N_7_H_3_ interface. *Φsb* is the hole Schottky barrier. (**b**) Calculated imaginary part of the dielectric function (in-plane polarization) for the graphene/g-C_12_N_7_H_3_ heterojunction with HSE06 methods.

The fourth strategy is forming graphene/g-C_12_N_7_H_3_ heterojunction. In this situation, the heterojunction is formed by a metal and a semiconductor. In fact, experiments have demonstrated that the graphene/g-C_3_N_4_ composite could harvest a broad range of visible light efficiently, i.e., it can potentially lead to an enhanced photocatalytic activity^50^. In the process of forming a heterojunction with graphene and g-C_12_N_7_H_3_, the interaction between two layers exerts a driving force, which leads to the electron transfer from the areas of graphene to the areas of g-C_12_N_7_H_3_ monolayer, while holes move in the opposite way. After a period of time, the charge redistribution in the graphene/g-C_12_N_7_H_3_ heterointerface reaches equilibrium, a built-in electric field is induced. As we have learned from semiconductor physics, since graphene is a semimetal and g-C_12_N_7_H_3_ is a semiconductor, the space charge region of the graphene/g-C_12_N_7_H_3_ heterointerface results in the formation of a Schottky barrier (*φ*_*sb*_). In Fig. 8(a), the band bending associated with the Schottky barrier height is expressed.

In order to further understand a built-in electric field and Schottky barrier, we analyzed the band alignment and calculated the Schottky barriers by employing the lineup method^51^,^52^. First of all, the work functions of the g-C_12_N_7_H_3_ and graphene monolayers were calculated, which are equivalent to the differences between the vacuum level and the Fermi energy. The work function of graphene is 4.54 eV with the hybrid DFT method, in good accord with measured values in the range of 4.3–4.6 eV^53^,^54^. The calculated work function of the g-C_12_N_7_H_3_ is 6.26 eV. The Schottky barrier of the graphene/g-C_12_N_7_H_3_ composite was then determined as the difference between the Fermi energy of the bilayer and the VBM energy in an isolated g-C_12_N_7_H_3_ monolayer, at the same time considering the interface dipole potential as detailed in the work by Shan *et al*.^51^. The calculated*φ*_*sb*_ is 1.05 eV for holes to diffuse from graphene to g-C_12_N_7_H_3_. Our results are consistent with previous studies^55^.

Thus when the material absorbs the light, the charge carriers are photoexcited, photogenerated holes in the valence band of g-C_12_N_7_H_3_ are trapped due to the Schottky barrier, whereas the photogenerated electrons can freely diffuse from the conduction band of g-C_12_N_7_H_3_ to graphene [see Fig. 8(a)]. Therefore, photoexcited charge carriers can be separated effectively at the graphene/g-C_12_N_7_H_3_ heterojunction, which leads to higher energy utilization efficiency and improves the photocatalytic performance^56^.

Meanwhile, we calculated imaginary part of the dielectric function for the graphene/g-C_12_N_7_H_3_ heterojunction, as shown in Fig. 8(b). The absorption spectrum of the heterojunction has been significantly expanded, indicating that the graphene/g-C_12_N_7_H_3_ composite could harvest a broad range of visible light efficiently. It should be noted that the enhanced visible light response is consistent with the most recent experimental observation at NIMS in Japan^57^. It also can be understood that graphene sheets act as conductive channels to efficiently separate the photogenerated charge carriers and to enhance the visible-light photocatalytic H_2_-production activity of g-C_12_N_7_H_3._

## Conclusions

In conclusion, we have investigated the stability of g-C_12_N_7_H_3_ through phonon dispersion relations and MD simulations. The X-ray diffraction spectra, which can be regarded as fingerprints to identify the g-C_12_N_7_H_3_ from other graphitic carbon nitride materials, have been simulated. Using first-principles calculations, we have systematically studied the electronic structure, band edge alignment, and optical properties for the g-C_12_N_7_H_3_. The results demonstrated that g-C_12_N_7_H_3_ is a new organocatalyst material for water splitting. In order to enhance the photocatalytic efficiency, we provided four strategies, i.e., multilayer stacking, raising N atoms, forming g-C_9_N_10_/g-C_12_N_7_H_3_ heterojunction, forming graphene/g-C_12_N_7_H_3_ heterojunction. Our theoretical results will encourage experimental finding of 2D metal-free organic materials as visible light photocatalysts. More importantly, we propose an effective strategy- forming heterojunction, which can improve the electron-hole separation. Our strategies will further promote the development of metal-free organic photocatalyst.

## Methods

Our first-principles calculations were performed within the framework of density-functional theory (DFT), which is implemented in the Vienna ab initio simulation package known as VASP^58^,^59^,^60^. The electron-electron interactions were treated using a generalized gradient approximation (GGA) in form of Perdew-Burke-Ernzerhof (PBE) for the exchange-correlation function^61^. The energy cutoff employed for plane-wave expansion of electron wavefunctions was set to 600 eV. The electron-ion interactions were described by projector-augmented-wave (PAW) potentials^62^,^63^. Four electrons for carbon (2s^2^2p^2^), five electrons for nitrogen (2s^2^2p^3^) and one electron for hydrogen (1s^1^) were treated as valence electrons. The supercells were repeated periodically on the x-y plane while a vacuum region of 15 Å was applied along the z-direction to avoid mirror interactions between neighboring images. The BZ integration was sampled on a grid of 7 × 7 × 1k-points. Structural optimizations were carried out using a conjugate gradient (CG) method until the remaining force on each atom was less than 0.001 eV/Å.

The X-ray diffraction (XRD) spectra were calculated according to the Bragg equation^64^,^65^. The expression of Bragg equation is 2d sin*θ* = *nλ*, where λ is the wavelength of X ray, and *n* is for any positive integer, also known as the diffraction series. In the calculation of the energy band, a more accurate Heyd–Scuseria–Ernzerhof (HSE) screened potential method^29^ was used. Van der Waals correction of the Grimme’s D2 scheme^66^ was considered to compute the non-covalent interaction of multilayer CTFs. The work function was calculated by: φ = V(*∞*) − *E*_*F*_, where V(*∞*) and *E*_*F*_ are the electrostatic potential in a vacuum region far from the neutral surface and the Fermi energy of the neutral surface system, respectively^67^. The vacuum level was taken as the reference in the calculations of band alignment. Using the Kramers–Kronig dispersion relation^68^, the optical absorption spectras were calculated from the imaginary part of the dielectric function.

## Additional Information

**How to cite this article**: Li, H. *et al*. Forming heterojunction: an effective strategy to enhance the photocatalytic efficiency of a new metal-free organic photocatalyst for water splitting. *Sci. Rep.* **6**, 29327; doi: 10.1038/srep29327 (2016).

## Supplementary Material

Supplementary Table S1

## Figures and Tables

**Table 1:** The work function (WF) and band gap (E_g_) of two-, three-, four-, five-, and six-layer 2D g-C_12_N_7_H_3_ computed with HSE06 methods.

Layers	1	2	3	4	5	6	n
WF(eV)	6.76	6.72	6.70	6.69	6.68	6.68	—
E_g_(eV)	3.24	2.91	2.77	2.69	2.64	2.61	2.61
ΔE_g_(eV)	0	0.33	0.47	0.55	0.60	0.63	0.63

The band gap differences (ΔE_g_) between the multilayers and monolayer g-C_12_N_7_H_3_ are shown.
