# Supplementary material for: Forming heterojunction: an effective strategy to enhance the photocatalytic efficiency of a new metal-free organic photocatalyst for water splitting
Source: Sci Rep. 2016 Jul 29;6:29327. doi: 10.1038/srep29327 (PMC4965868; doi:10.1038/srep29327)
Supplement: Supplementary Table S1 [file srep29327-s1.pdf]

Supplementary Information for

Forming heterojunction: an effective strategy to enhance the photocatalytic efficiency of a new metal-free organic photocatalyst for water splitting

Hengshuai Li,[*,1,2] Haiquan Hu,[3] Chunjiang Bao,[1] Feng Guo,[3] Xiaoming Zhang,[2] Xiaobiao Liu,[2] Juan Hua,[2] Jie Tan,[2] Aizhu Wang,[2] Hongcai Zhou,[2] Bo Yang,[2] Yuanyuan Qu,[2] Xiangdong Liu,[2]

[1]*School of Mechanical & Automotive Engineering, Liaocheng University, Liaocheng, 252059, China*

[2]*School of Physics and State Key Laboratory of Crystal Materials, Shandong University, Jinan 250100, China*

[3]*School of Physics Science and Information Technology, Liaocheng University, Liaocheng, 252059, China*

Supplementary Table

Table S1 The work function (WF) and band gap (E_g) of two-, three-, four-, five-, and six-layer 2D g-$C_{12}N_7H_3$ computed with PBE function. The band gap differences (ΔE_g) between the multilayers and monolayer g-$C_{12}N_7H_3$ are shown.

Layers	1	2	3	4	5	6	n
WF(eV)	6.26	6.20	6.20	6.18	6.13	6.13	----
E_g(eV)	2.30	1.98	1.84	1.77	1.72	1.69	1.69
ΔE_g(eV)	0	0.32	0.46	0.53	0.58	0.61	0.61
